# Potential Metabolite Markers for Pancreatic Cancer Identified by Metabolomic Analysis of Induced Cancer-Associated Fibroblasts

**DOI:** 10.3390/cancers14061375

**Published:** 2022-03-08

**Authors:** Yoshihiro Miyazaki, Nobuhito Mori, Yuka Akagi, Tatsuya Oda, Yasuyuki S. Kida

**Affiliations:** 1 Department of Gastrointestinal and Hepato-Biliary-Pancreatic Surgery, Faculty of Medicine, University of Tsukuba, Tsukuba 305-8575, Ibaraki, Japan; miyazaki.yoshihiro.su@ms.hosp.tsukuba.ac.jp (Y.M.); tatoda@md.tsukuba.ac.jp (T.O.); 2 Cellular and Molecular Biotechnology Research Institute, National Institute of Advanced Industrial Science and Technology (AIST), Tsukuba 305-8565, Ibaraki, Japan; n-mori@aist.go.jp (N.M.); y-akagi@aist.go.jp (Y.A.); 3 Advanced Photonics and Biosensing Open Innovation Laboratory, National Institute of Advanced Industrial Science and Technology (AIST), Tsukuba 305-8565, Ibaraki, Japan; 4 School of Integrative and Global Majors, University of Tsukuba, Tsukuba 305-8575, Ibaraki, Japan

**Keywords:** cancer-associated fibroblasts, tumor microenvironment, pancreatic cancer, intracellular metabolism, glycolysis, oxidative phosphorylation, cell differentiation

## Abstract

**Simple Summary:**

Fibroblasts in normal tissues conduct energy metabolism via oxidative phosphorylation (OXPHOS). However, cancer-associated fibroblasts (CAFs) produce energy (i.e., ATP) via glycolysis. Nonetheless, whether intracellular metabolism transitions from OXPHOS to glycolysis when normal tissue fibroblasts differentiate into CAFs remains to be determined. Here, we established an experimental system and induced the in vitro differentiation of mesenchymal stem cells to CAFs and performed detailed metabolomic and RNA sequencing analyses. We found that the intracellular metabolic pathway was reprogrammed to the glycolytic pathway when mesenchymal stem cells were co-cultured with pancreatic cancer cells. Furthermore, we identified CAF-specific metabolites that were expressed post reprogramming. These metabolites have also been observed in pancreatic cancer mouse models, suggesting their potential as cancer biomarkers.

**Abstract:**

Cancer-associated fibroblasts (CAFs) in the tumor microenvironment perform glycolysis to produce energy, i.e., ATP. Since the origin of CAFs is unidentified, it is not determined whether the intracellular metabolism transitions from oxidative phosphorylation (OXPHOS) to glycolysis when normal tissue fibroblasts differentiate into CAFs. In this study, we established an experimental system and induced the in vitro differentiation of mesenchymal stem cells (MSCs) to CAFs. Additionally, we performed metabolomic and RNA-sequencing analyses before and after differentiation to investigate changes in the intracellular metabolism. Consequently, we discovered that OXPHOS, which was the primary intracellular metabolism in MSCs, was reprogrammed to glycolysis. Furthermore, we analyzed the metabolites in pancreatic tumor tissues in a mice model. The metabolites extracted as candidates in the in vitro experiments were also detected in the in vivo experiments. Thus, we conclude that normal tissue fibroblasts that differentiate into CAFs undergo a metabolic reprogramming from OXPHOS to glycolysis. Moreover, we identified the CAF-specific metabolites expressed during metabolic reprogramming as potential future biomarkers for pancreatic cancer.

## 1. Introduction

The most common solid tumors, such as pancreatic cancer, have a large number of stromal cells. Indeed, the stroma forms a thickened extracellular matrix that provides a unique environment, such as a hypoxic environment by inhibiting blood vessel formation in a tumor. The thickened extracellular matrix acts as a barrier that protects cancer cells from attack by the immune system and anticancer drugs, drastically reducing the response rate of patients to cancer therapy [1,2,3].

The tumor microenvironment (TME) comprises cancer cells surrounded by a variety of stromal cells, including cancer-associated fibroblasts (CAFs), tumor-associated macrophages, and infiltrating immune cells. These stromal cells interact with cytokines and metabolites and create an environment that promotes the aggressive growth of cancer cells [4,5].

Notably, CAFs are the most abundant cells in the TME. Studies have demonstrated that CAFs are produced upon the epithelial-to-mesenchymal transition of cancer cells and are derived from fibroblasts localized in tissues [6,7], and mesenchymal stem cells (MSCs) derived from the bone marrow [8] or adipose tissue [9,10]. However, their origin has not been determined in human tumors despite their presence being determined in tumorigenesis experiments in vivo. In fact, most CAFs in the TME do not have the genetic mutations that are typically observed in cancer cells. Recently, researchers have expressed increasing interest in how normal cells, such as MSCs, adapt to the TME and differentiate into CAFs that support carcinogenesis; indeed, much research has been done to elucidate this mechanism [11]. Drug discovery-based research targeting the inhibition of CAF-mediated stromal support to kill cancer cells has also been attracting attention [12].

Hypoxia and low nutrient supply make the TME an unfavorable environment for cell proliferation. Interestingly, cancer cells overcome this disadvantage by expressing hypoxia-responsive genes, thereby proliferating rapidly [13]. Moreover, they counteract this unfavorable environment of the TME by maintaining a special intracellular metabolic state called the Warburg effect, and also owing to powerful genetic mutations, such as those in *KRAS* and *TP53* [14,15]. However, how CAFs survive adverse conditions, such as hypoxia and low nutrient supply in the TME has not yet been well studied. Inhibition of several gene functions in CAFs has suggested that glycolysis is upregulated in CAF, thereby increasing ATP production. Moreover, CAFs have been demonstrated to release metabolites that are necessary for the survival and functioning of cancer cells [16,17]. This is called the reverse Warburg effect and is considered to be one of the primary mechanisms through which CAFs propagate cancer cell survival. However, metabolic shifts in a normal cell, such as the upregulation of glycolysis and downregulation of oxidative phosphorylation (OXPHOS), has not been studied yet. This is because progenitor cells of CAFs are as yet unidentified, thereby making it difficult to experimentally induce cell differentiation to CAFs.

Previously, we have demonstrated that adipose-derived MSCs (AD-MSCs) can differentiate into CAFs in vitro and in vivo, and can reproduce a robust TME that is frequently observed in pancreatic cancer [10,18]. These studies revealed that AD-MSCs are progenitors of CAFs; they morphologically transform into CAFs, exhibit alterations in their gene expression, and acquire heterogeneity. We demonstrated that myoblastic CAF (myCAF) is induced upon direct co-culture of cancer cells and AD-MSCs, and inflammatory CAF (iCAF) is induced upon indirect co-culture of these cells [18]. In the present study, we performed in vitro metabolomic analysis in induced CAFs to understand the metabolic shift that takes place in CAFs. We discovered a prominent metabolic shift towards glycolysis accompanied with downregulated OXPHOS, and identified novel metabolites that were produced during cell differentiation to CAFs.

## 2. Materials and Methods

### 2.1. Cells and Culture Conditions

The immortalized human AD-MSC cell line ASC52telo (ATCC SCRC-4000) and the human pancreatic cancer cell line Capan-1 (ATCC HTB-79) were used and cultured as previously described [10,18]. Briefly, these cells were maintained in Dulbecco’s Modified Eagle’s Medium (DMEM; FUJIFILM Wako Pure Chemical Corp., Osaka, Japan) supplemented with 20% fetal bovine serum (FBS), 1% nonessential amino acids, and 1% streptomycin–penicillin at 37 °C in a humidified atmosphere containing 5% CO_2_. Additionally, the KPC cell line derived from the KPC (LSL-*KRAS*^G12D/+^; LSL-*TP53*^R172H/+^; *PDX1*-CRE) mice [19] was maintained in DMEM supplemented with 10% FBS and 1% streptomycin–penicillin at 37 °C in a humidified atmosphere containing 5% CO_2_. To establish the KPC cell line isolated from KPC mice, pancreatic tumor was minced, and two or three 2 mm tumor pieces were plated onto a dish in the medium. The dishes were then incubated under 5% CO_2_, 20% O_2_, at 37 °C. After a couple days of incubation, cancer cells grew around the tumor fragments, and the tumor fragments were removed. When a sufficient number of cells were observed, the cells were passaged or stored. Under these culture conditions, cancer cells grew selectively, while the other cells including CAFs were depleted after a few passages. We confirmed that these cells contained only KPC cells and no other cells by flowcytometry before further analysis. Subsequently, the AD-MSCs and Capan-1 cells were labeled with green fluorescent protein (GFP) and red fluorescent protein (RFP) by lentiviral transduction, respectively. For this purpose, we seeded 293LTV cells (LTV-100; Cell Billabs, Inc., San Diego, CA, USA) into a 6-well plate and co-transfected them with pLKO.1-puro eGFP (Sigma-Aldrich, St. Louis, MO, USA), psPAX2 was a gift from Didier Trono (Addgene plasmid # 12260; http://n2t.net/addgene:12260, accessed on 17 January 2022; RRID:Addgene_12260), and pMD2.G was a gift from Didier Trono (Addgene plasmid # 12259; http://n2t.net/addgene:12259, accessed on 17 January 2022; RRID:Addgene_12259) at a ratio of 2:1.5:1.2 μg/well. Post 24 h, the viral supernatant was collected and filtered through 0.45 μm membranes. Thereafter, AD-MSCs were infected with this filtrate in the presence of polybrene (10 μg/mL) for 24 h. Thereafter, puromycin was added to the medium for the selection of GFP-positive cells. Once we identified AD-MSCs that expressed GFPs, we confirmed that these cells still retained their original characteristics, such as auto-differentiation, senescence, or weak stemness.

### 2.2. In Vitro Co-Culture Assay

Co-culturing of AD-MSCs and Capan-1 cells was performed as described previously [18]. In this regard, AD-MSCs and Capan-1 cells were co-cultured under two different conditions: direct and indirect transwell co-culture. In the direct co-culture method, 4 × 10^5^ AD-MSCs and 4 × 10^5^ Capan-1 cells were mixed and seeded in 6-well culture plates. In contrast, in indirect transwell co-culture, 4 × 10^5^ AD-MSCs were seeded in the lower compartment of the transwell membrane, whereas 4 × 10^5^ Capan-1 cells were seeded in the upper compartment (Falcon Permeable Support for 6-well plates with 3.0 μm translucent high-density PET membrane #353092; Corning Inc., Corning, NY, USA).

### 2.3. Immunofluorescence (IF) Staining of Cells in Monocultures and Co-Cultures

We fixed AD-MSCs and Capan-1 cells with 100% methanol for 10 min at −20 °C. Subsequently, we washed them three times for 5 min each with an IF buffer solution (10 × stock: 38.0 g NaCl, 9.38 g Na_2_HPO_4_, 2.07 g NaH_2_PO_4_, 2.5 g NaN_3_, 5.0 g bovine serum albumin (BSA), 10 mL Triton X-100, and 2.5 mL Tween-20 in 500 mL PBS). Thereafter, the cells were treated with a blocking solution (3% BSA in 1 × IF washing solution) for 30 min. They were then incubated for 1 h at 25 °C with a mouse SMA antibody (1:400, ab7817; Abcam, Cambridge, UK), rabbit anti IL-6 antibody (1:200, ab6672; Abcam), or rabbit anti-rat vimentin antibody (1:400, #280618; R&D Systems Inc., Minneapolis, MN, USA) in the 1 × IF buffer. Post this incubation, the cells were washed three times with the 1 × IF buffer. They were subsequently incubated for 1 h with Alexa Fluor 488 goat anti-rabbit IgG antibody (Invitrogen, Carlsbad, CA, USA), Alexa Fluor 568 goat anti-rat IgG antibody (Invitrogen), and Cy5 goat anti-rabbit IgG antibody (Invitrogen), all diluted to 1:400 in the 1 × IF buffer solution. The cell nuclei were counterstained with Hoechst 33342 (Thermo Fisher Scientific, Waltham, MA, USA) and thereafter were washed thrice with the 1 × IF buffer. The slides were mounted and visualized under a fluorescence microscope (BZ-710, Keyence, Osaka, Japan).

### 2.4. In Vitro Macropinocytosis Assay

We performed an in vitro macropinocytosis assay as previously described [20]. Briefly, 1 × 10^5^ cells were plated onto glass coverslips in 6-well plates for five days. We then incubated the cells for 30 min at 37 °C with 70-kDa fluoresceine isothiocyanate (FITC)-dextran (Sigma) directly added to the culture media at a final concentration of 1 mg/mL. Subsequently, we assessed the macropinocytic uptake of cells, and rinsed the cells five times on ice with ice-cold PBS. Thereafter, the cells were fixed with 3.7% formaldehyde and their nuclei were counterstained with DAPI. Eventually, the coverslips were mounted onto glass slides using an aqua-poly/mount (Polysciences, Inc., Warrington, PA, USA). Fluorescent images were captured using a fluorescence microscope (BZ-710; Keyence). Notably, each experimental condition was performed in triplicates.

### 2.5. Mouse Models and In Vivo Experiments

We purchased eight-week-old female C57BL/6J wild mice from Japan CLEA Inc. (CLEA Japan, Tokyo, Japan) for our experiments. We bred and housed the mice under specific pathogen-free conditions at the Animal Center of AIST and the University of Tsukuba. We developed a xenograft model by subcutaneously transplanting 2 × 10^6^ KPC cells into the mice (KPC xenograft model). Thereafter, we sacrificed the mice on day 28 and excised all subcutaneous tumors.

All invasive procedures were performed under inhalation anesthesia using isoflurane. Mice were euthanized by cervical dislocation following inhalation of the anesthesia. All animal experiments and procedures were approved by and performed in compliance with the guidelines of Institutional Animal Care and Use Committee of the respective institutes of AIST (A2020-310) and the Ethics Committee of the University of Tsukuba (19-028). The study was conducted in accordance with the Animal Research Reporting in vivo Experiments (ARRIVE) guidelines [21].

### 2.6. Immunohistochemical Tissue Staining

All the staining protocols were performed on 2 µm thick mouse tissue sections. Hematoxylin and eosin (HE) and Masson’s trichrome (MT) staining were performed according to standard protocols [10]. We performed immunohistochemistry (IHC) by first deparaffinizing the sections, following which we performed antigen retrieval at 121 °C in an autoclave for 10 min in a 10 mM sodium citrate buffer (pH 6.0). We then treated the sections with a 3% H_2_O_2_ solution (Envision Plus System; Dako, Santa Clara, CA, USA) to inhibit any endogenous peroxidases. Rabbit polyclonal LIF antibody (1:500, ab113262; Abcam) was used for IHC. The labeled antigens were visualized by chromogen 3,30-diaminobenzidine tetrahydrochloride; hematoxylin was used as a nuclear counterstain. Eventually, the slides were observed under a fluorescence microscope (BZ-710; Keyence).

### 2.7. Metabolomic Analysis

Monocultured and co-cultured Capan-1 cells and AD-MSCs (each group *n* = 3) and KPC xenograft mice tumors (*n* = 3) were analyzed by metabolomics (Human Metabolome Technologies (HMT) Inc., Tsuruoka, Japan) [22,23,24]. Firstly, frozen cells or mouse tumor samples were transferred into 500 μL of methanol containing 50 mM of an external standard. Thereafter, we homogenized the cells five times at 200× *g* for 120 s by BMSM10N21 (BMS, Tokyo, Japan). Following this, we added and mixed 500 μL of chloroform and 200 μL of ultrapure water to the homogenate, and subsequently centrifuged the mixture at 2300× *g* for 5 min at 4 °C. The resultant aqueous phase was subjected to ultrafiltration using a Millipore Ultrafree-MC PLHCC HMT Centrifugal Filter Device, 5 kDa (Millipore, Billerica, MA, USA). The filtrates were then dried and dissolved in 50 μL of ultrapure water. Subsequently, the samples were subjected to capillary electrophoresis time-of-flight mass spectrometry (CE-TOFMS) in the Agilent CE-TOFMS system (Agilent Technologies, Santa Clara, CA, USA) at 4 °C. The detected peaks were aligned according to the *m/z* values and normalized migration times. Furthermore, the peaks were mean-centered and scaled using their standard deviations on a per-peak basis as a pretreatment. Following the application of autoscaling, we conducted principal component analysis (PCA) and hierarchical clustering analysis using SampleStat v3.14 (HMT Inc., Tsuruoka, Japan) and PeakStat v3.18 (HMT Inc.). In the PCA, score plots of the first and second principal components were generated. Additionally, we generated heat maps by coloring the data values across their value ranges. The relative area of each peak was calculated and used for comparison among the four groups.

### 2.8. RNA Extraction and Quantitative Real-Time PCR (qPCR)

We extracted total RNA from the cells using the TRI Reagent (Molecular Research Center, Inc., Cincinnati, OH, USA), according to the manufacturer’s instructions. Subsequently, 500 ng of the extracted RNA was converted to cDNA using RevaTra Ace reverse-transcription reagents (TOYOBO, Osaka, Japan), as per the manufacturer’s instructions. Next, we performed qPCR using commercially available gene-specific PrimeTime qPCR probes (listed below; purchased from Integrated DNA Technologies, Coralville, CA, USA) and 2 9 Thunderbird Probe qPCR mix (TOYOBO), in accordance with the manufacturer’s instructions. The following PrimeTime qPCR probes were used (Hs, Human probes): C-X-C motif chemokine ligand 1 (*CXCL1*), Hs.PT.58.39039397; glyceraldehyde-3-phosphate dehydrogenase (*GAPDH*), Hs.PT.39a.22214836; actin alpha 2, smooth muscle (*ACTA2*), Hs.PT.56a.2542642; Interleukin 6 (*IL6*), Hs.PT.58.40226675; leukemia inhibitory factor (*LIF*), Hs.PT.58.27705899, and connective tissue growth factor (*CTGF*), Hs.PT.58.14485164.g. Expression levels of the target genes were normalized to that of *GAPDH*.

### 2.9. RNA-Sequencing (RNA-Seq)

We evaluated the difference in the gene expression levels of AD-MSCs and AD-CAFs by performing RNA-seq as previously reported [10,18]. Briefly, total RNA was extracted from the cells using the TRI Reagent (Molecular Research Center, Inc., Cincinnati, OH, USA). Thereafter, library preparation and sequencing were conducted using the Truseq library prep kit and NovaSeq 6000 (Illumina, San Diego, CA, USA). The data acquired from two biological replicates for each cell type were analyzed using STAR (2.7.1a, Cold Spring Harbor Laboratory, Cold Spring Harbor, NY, USA) [25], RSEM (1.3.1, University of Wisconsin-Madison, Madison, WI, USA) [26], and edgeR (3.30.3, Walter and Eliza Hall Institute of Medical Research, Victoria, Australia) [27]. Subsequently, we obtained the normalized counts (trimmed mean of M values) and identified the differentially expressed genes that satisfied the condition |log2 (fold-change)| ≥ 1 and false discovery rate < 0.05. Raw sequences in the FASTQ format were deposited at the DNA Data Bank of Japan (DDBJ; accession numbers DRR231745–DRR231748).

### 2.10. Statistical Analysis

Data are represented as the mean ± standard deviation (SD) unless otherwise noted. We analyzed the data among groups of three or more groups by one-way analysis of variance followed by post hoc Tukey tests with two-tailed distribution. Furthermore, the student’s *t*-test was used to compare data between the control and experimental groups. Statistical significance was set at *p* < 0.05. All calculations were performed using the GraphPad Prism software or EZR (Saitama Medical Center, Jichi Medical University, Saitama, Japan), which is a graphical user interface of the R software (R Foundation for Statistical Computing, Vienna, Austria) [28]. Of note, EZR is a modified version of R Commander designed for statistical functions that are frequently used in biostatistics. Additionally, the SD of the data are represented as error bars in the figures.

## 3. Results

### 3.1. AD-MSCs Co-Cultured with Capan-1 Cells Are CAF-Progenitors That Are Capable of Reacting with Cancer Cells

**Figure 1:**
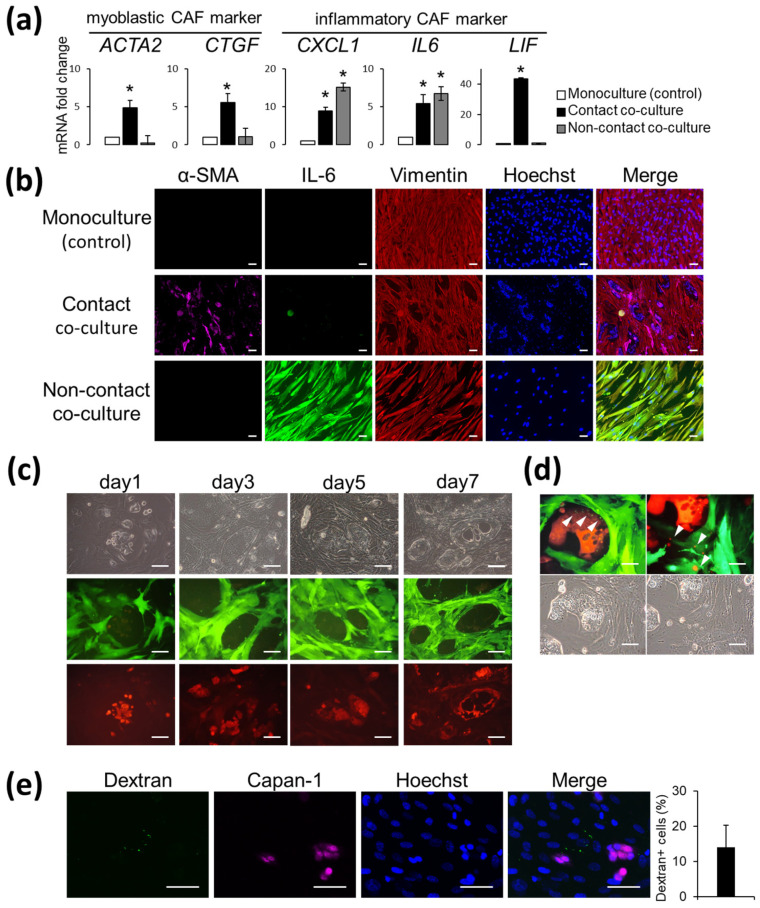
Adipose-derived mesenchymal stem cells (AD-MSCs) differentiated into adipose-derived cancer-associated fibroblasts (AD-CAFs) upon co-culture and interaction with Capan-1 cells: (**a**) qPCR analysis of myoblastic cancer-associated fibroblast (CAF) markers or inflammatory CAF markers in three culture conditions. Results are presented as the mean ± standard deviation of three biological replicates. * *p* < 0.05, unpaired Student’s *t*-test; (**b**) Representative immunofluorescent image of AD-MSCs co-cultured with/without Capan-1 cells and stained for αSMA (magenta), IL-6 (green), and vimentin (red). Counterstain: Hoechst 33342 (blue). Scale bar: 100 μm; (**c**) Representative image of direct contact co-culture of green fluorescent protein (GFP)-tagged CAFs with red fluorescent protein (RFP)-tagged Capan-1 cells on days 1, 3, 5, and 7. Scale bar: 100 μm; (**d**) Magnified images of direct contact co-culture of GFP-tagged CAFs with RFP-tagged Capan-1 cells on day 7. Arrowheads indicate vesicles formed by interaction of AD-MSCs with Capan-1 cells. Scale bar: 50 μm; (**e**) Representative fluorescent image confirming macropinocytosis in the co-culture of AD-MSCs and Capan-1 cells (magenta) after the addition of FITC-dextran and quantification of dextran uptake by cells. Counterstain: Hoechst 33342 (blue). Scale bar: 50 μm.

We generated CAFs derived from AD-MSCs (AD-CAFs) by two methods: direct contact and indirect non-contact transwell co-culture with the Capan-1 cell line. Our qPCR analysis confirmed that these two methods generated AD-CAFs by two distinct mechanisms of differentiation (Figure 1a). For instance, AD-CAFs generated by contact co-culture exhibited increased expression of the myoblast markers *ACTA2* and *CTGF*. On the other hand, AD-CAFs generated by the non-contact co-culture had upregulated expression of cytokine-related genes, such as *CXCL1*, *IL6*, and *LIF* (Figure S1). These alterations were verified by immunostaining (Figure 1b). Moreover, the αSMA protein was more prominently expressed in the direct co-culture than in the non-contact co-culture. Notably, IL6-positive cells were rarely observed in this approach. In contrast, the number of IL6-positive cells was greatly increased and αSMA-positive cells were not observed when cells were cultured via the non-contact transwell co-culture. However, vimentin, a fibroblast marker, had uniform expression in both the co-cultures, confirming that the analyzed cells were indeed CAFs. Next, we examined whether co-culturing GFP-transfected AD-MSCs with RFP-transfected Capan-1 cells resulted in cell-cell interactions. Indeed, we observed vesicles after three days of contact co-culture (Figure 1c), and the presence of green vesicles in Capan-1 cells or red vesicles in CAFs was confirmed under a strong magnification (Figure 1d). Furthermore, we observed the presence of several FITC-positive vesicles when dextran-FITC was added to the co-culture media (Figure 1e). These observations verified that AD-MSCs differentiate into AD-CAFs when co-cultured with Capan-1 cells. Therefore, we used AD-MSCs as progenitors of CAFs for subsequent metabolomic analysis. Notably, we used AD-MSCs maintained in non-contact co-cultures in consideration of the time required for sample collection and to prevent contamination between AD-MSCs and Capan-1 cells.

### 3.2. AD-CAFs Undergo a More Drastic Metabolic Transformation Than Capan-1, as Discovered by a Global Metabolomic Analysis

**Figure 2:**
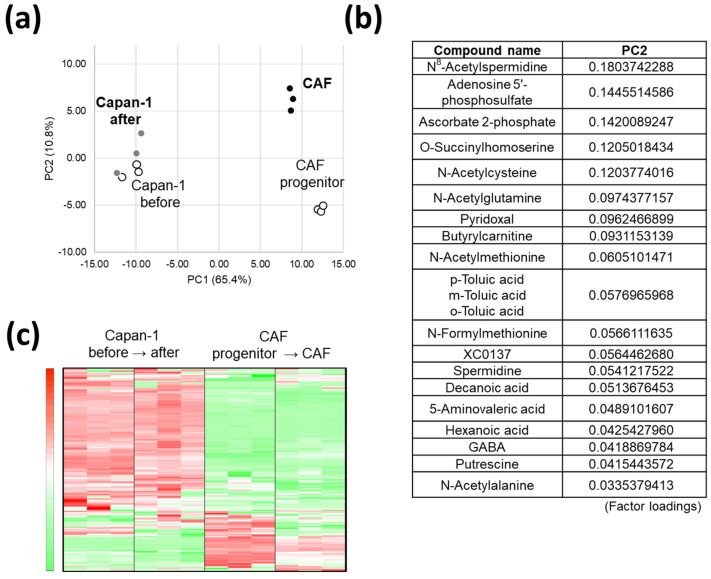
Global trend of metabolite changes in cancer cells (Capan-1 cells) and cancer-associated fibroblasts (CAFs): (**a**) Principal component analysis (PCA) of metabolomic datasets of Capan-1 cells and CAFs grown in a non-contact transwell co-culture; (**b**) The metabolites involved in PC2-axis and their contribution rates; (**c**) Heat map comparing altered metabolites between Capan-1 cells (*n* = 3) and CAFs (*n* = 3).

We performed metabolomic analyses of AD-MSCs (progenitors of CAFs), AD-CAFs, and Capan-1 cells before and after co-culture by CE-TOFMS (Figure 2a). The results of our PCA are presented in Figure 2a. Remarkably, we observed that the metabolite profile of AD-MSCs was significantly different from AD-CAFs. The metabolites involved in the PC2-axis in Figure 2a, and their contribution rates are represented in Figure 2b. Interestingly, these include metabolites that are involved in polyamine metabolism, but not those directly involved in major intracellular metabolic pathways, such as glycolysis and OXPHOS. Since these identified metabolites have not been well-studied, they are possible novel biomarkers of CAF-abundant tumors; we have discussed this matter in detail in later sections. On the other hand, lesser metabolite changes were observed in Capan-1 than in CAF. A heat map of the metabolite changes in each cell type is shown in Figure 2c. Overall, metabolites that were abundant in Capan-1 cells were scarce in CAFs, and conversely, metabolites that were abundant in CAFs were scarce in Capan-1 cells. This held true for metabolites that were highly abundant in Capan-1 cells both before and after co-culturing but were almost absent in CAFs both before and after co-culturing. These results indicate that each cell type had its own specific metabolite profile. Remarkably, only 36 metabolites exhibited altered abundance in Capan-1 cells; while 12 (33%) were increased in number, 24 (24%) were decreased. In contrast, 86 metabolites were altered in CAFs, of which 67 (78%) were downregulated. Thus, the metabolite changes observed in CAFs were larger than those observed in Capan-1 cells; notably, these changes indicated a decrease in abundance of the metabolites.

### 3.3. AD-CAFs Exhibit Upregulated Glycolytic Metabolism

**Figure 3:**
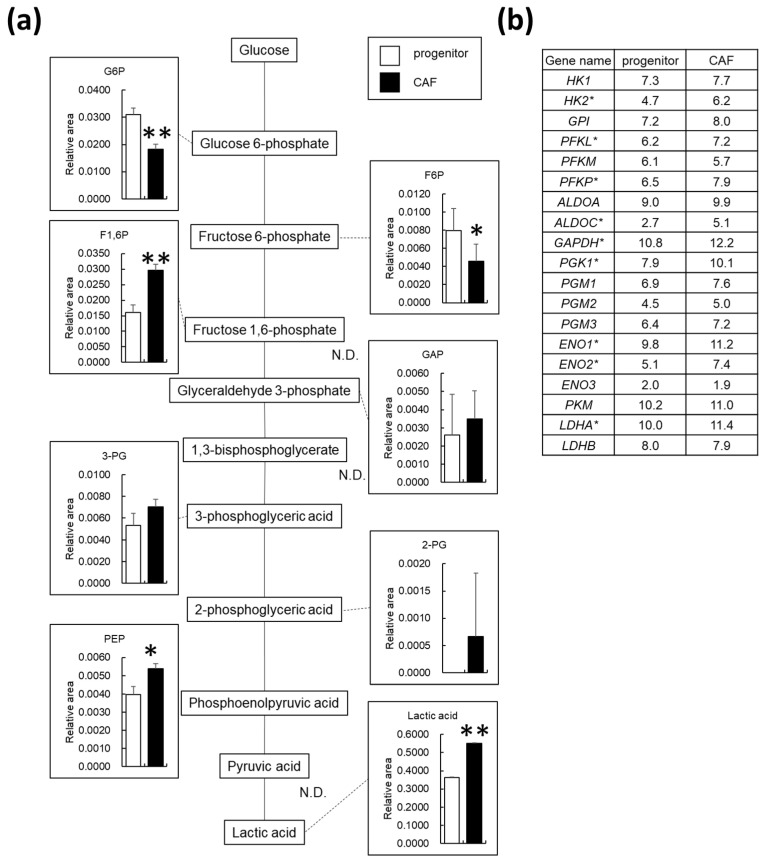
Changes in metabolites involved in glucose metabolism in the cancer-associated fibroblast (CAF) progenitors and CAFs: (**a**) Graphical representation of relative metabolite changes between CAF progenitors and CAFs as determined by capillary electrophoresis time-of-flight mass spectrometry. N.D. indicates not detected. ** *p* < 0.01, * *p* < 0.05; (**b**) List of genes involved in glycolysis and their expression levels in CAF progenitors and CAFs. Log2 normalized counts per million values. Asterisks (*) indicate differentially expressed genes.

We examined the changes in intracellular metabolism by comparing the metabolite profiles between AD-CAFs and AD-MSCs. Figure 3a depicts changes in the glycolytic metabolites, including glucose. While the levels of glucose 6-phosphate were substantially decreased upon differentiation of AD-MSCs to AD-CAFs, those of fructose 1,6-phosphate and phosphoenolpyruvic acid were increased by 1.9 and 1.4 folds, respectively. In addition, the aerobic glycolysis metabolite lactate was significantly increased (1.5 fold) in AD-CAFs, indicating that these cells undergo a metabolic shift towards glycolysis. We further validated this metabolic shift by analyzing altered gene expression levels by RNA-seq. The list of genes involved in glycolysis and their expression levels are displayed in Figure 3b. Consequently, we identified *HK2*, *PFKL*, *PFKP*, *ALDOC*, *GAPDH*, *PGK1*, *ENO1*, *ENO2*, and *LDHA* as differentially expressed genes (i.e., upregulated or downregulated gene expression). However, our pathway analysis did not reveal any significant changes, and the metabolic shift to glycolysis was not supported by our gene expression analysis.

### 3.4. OXPHOS Is Downregulated in AD-CAFs

**Figure 4:**
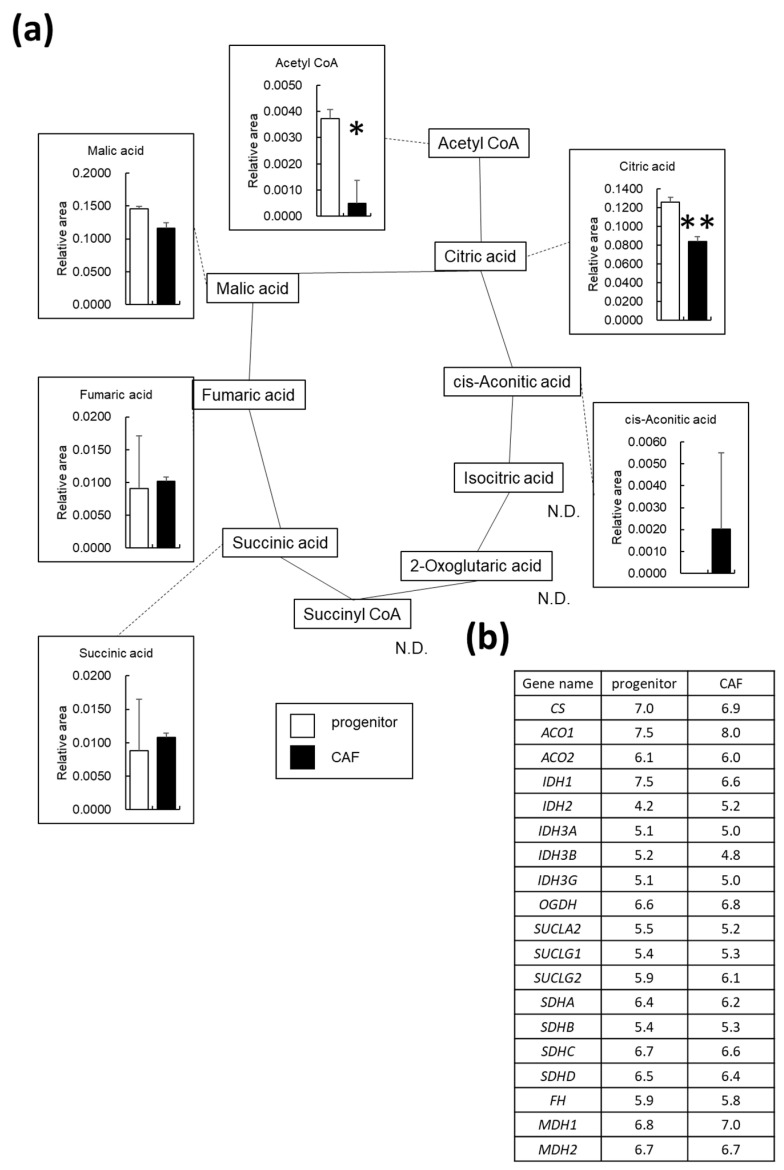
Changes in metabolites related to the tricarboxylic acid (TCA) cycle in the cancer-associated fibroblast (CAF) progenitors and CAFs: (**a**) Graphical representation of relative metabolite changes between CAF progenitors and CAFs, as determined by capillary electrophoresis time-of-flight mass spectrometry. N.D. indicates not detected. ** *p* < 0.01, * *p* < 0.05; (**b**) List of genes involved in the TCA cycle and their expression levels. Log2 normalized counts per million values. Asterisks (*) indicate differentially expressed genes.

Changes in the metabolites associated with the tricarboxylic acid (TCA) cycle are shown in Figure 4a. In this cycle, acetyl-CoA is synthesized from pyruvate and is converted to citric acid, and ATP is produced during reactions involving cis-aconitic acid, 2-oxoglutaric acid, and malic acid. Notably, acetyl-CoA (−7.4 fold), citric acid (−1.5 fold), and malic acid (−1.3 fold) were observed to be considerably downregulated in AD-CAFs. Indeed, we determined that downregulation of OXPHOS was associated with upregulation of glycolysis in AD-CAFs. The list of genes involved in OXPHOS and their expression levels are presented in Figure 4b. Remarkably, we observed no significant changes in the mRNA expression levels of these genes. Thus, the metabolic shift towards glycolysis in the AD-CAFs was not verified by changes in the gene expression levels.

### 3.5. Polyamine Metabolism Is Altered in AD-CAFs

**Figure 5:**
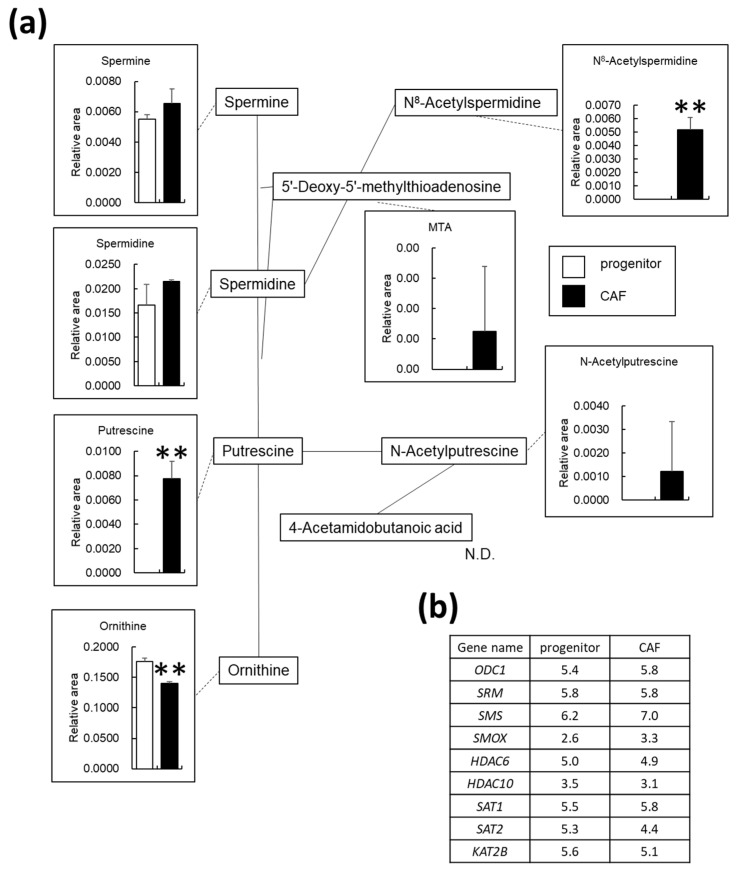
Changes in metabolites related to polyamine metabolism in cancer-associated fibroblasts (CAF) progenitors and CAFs: (**a**) Graphical representation of relative metabolite changes between CAF progenitors and CAFs, as determined by capillary electrophoresis time-of-flight mass spectrometry. N.D. indicates not detected. ** *p* < 0.01; (**b**) List of genes involved in polyamine metabolism and their expression levels. Log2 normalized counts per million values. There were no differentially expressed genes.

Polyamines are involved in various physiological functions, such as cell division and proliferation, and nucleic acid and protein synthesis; studies have also determined their roles in cancer cells [29,30,31]. Since we identified several polyamine metabolites in the PC2 axis in the PCA analysis (Figure 1), we also analyzed the polyamine synthesis pathway. Figure 5a presents changes in ornithine synthesized from arginine and those observed in metabolites involved in the polyamine synthesis pathway. We observed that ornithine, the starting point of this pathway, was significantly downregulated by 1.3-fold in the AD-CAFs. Moreover, while putrescine, N8-acetylspermidine, N-acetylputrescine, and 5′-deoxy-5′-methylthioadenosine were not detected in AD-MSCs, they were detected in AD-CAFs. A list of genes involved in the polyamine synthesis pathway and their expression levels are listed in Figure 5b. Since we observed no significant changes in the gene expression levels, the metabolic shift towards glycolysis was not supported by our gene expression analysis.

### 3.6. Certain Metabolites Are Uniquely Present in AD-CAFs

**Figure 6:**
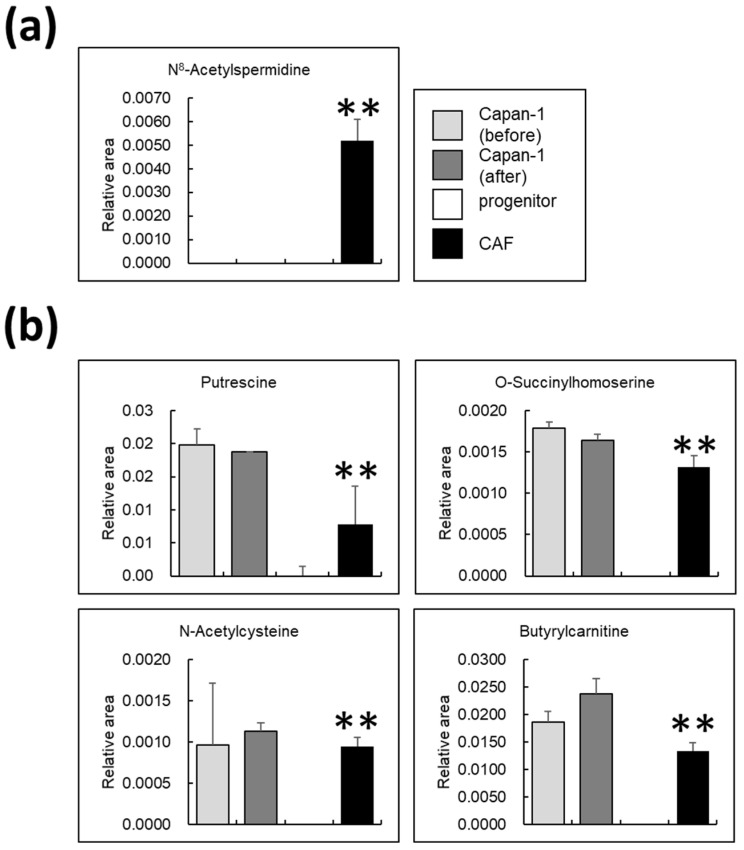
Metabolites specifically detected in cancer-associated fibroblasts (CAFs) alone and not CAF progenitors: (**a**) Amount of N8-acetylspermidine in all samples. N8-acetylspermidine was detected only in CAFs. ** *p* < 0.01; (**b**) Four metabolites detected solely in CAFs and not in the progenitors. ** *p* < 0.01.

Spermidine is acetylated at the N8 position by N-acetyltransferase in the cell nucleus. Subsequently, it is transported to the cytoplasm, where it is deacetylated by metal-dependent N8-acetylspermidine deacetylase, also called polyamine deacetylase, and N8-acetylspermidine is synthesized [32,33]. Alterations in the levels of N8-acetylspermidine in all the samples, including Capan-1 cells, are presented in Figure 6a. Interestingly, we detected this metabolite only in AD-CAFs. Therefore, we deduced that it is possibly a specific biomarker for CAF-rich cancers. On the other hand, the metabolites that were not detected in CAF progenitors were detected in AD-CAF, although they were not changed in Capan-1 before and after co-culture, as shown in Figure 6b. One such metabolite was the polyamine putrescine. Indeed, putrescine was detected in both AD-CAF and Capan-1. However, given that tumors in vivo are a mixture of cancer cells and CAFs, and the total amount of putrescine was increased after co-culture, putrescine may be a specific biomarker. Similarly, N-acetylcysteine, O-succinylhomoserine, and butyrylcarnitine could be regarded as specific metabolites of pancreatic cancer, as they were detected specifically in AD-CAFs.

### 3.7. Metabolites Unique to AD-CAFs Are Also Expressed in Mouse Pancreatic Cancer Models

**Figure 7:**
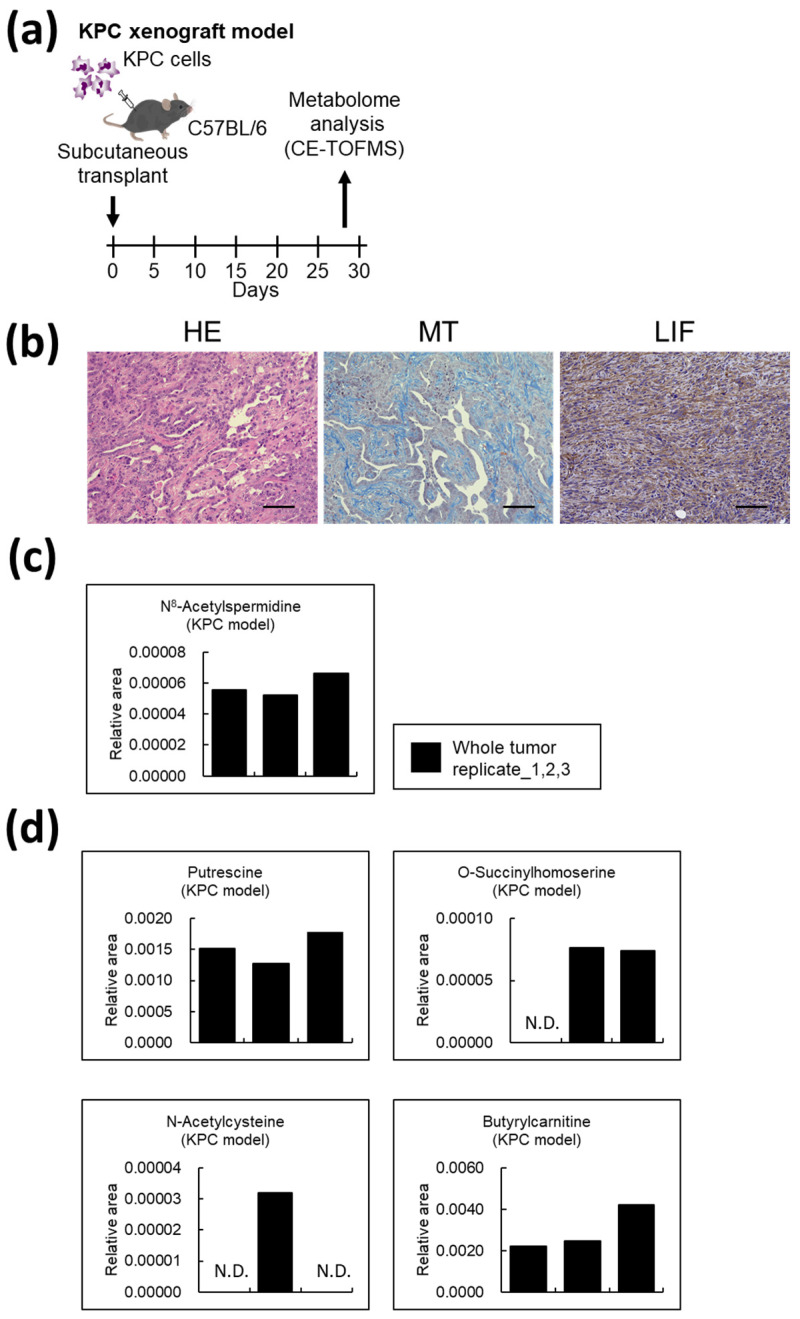
Unique metabolites detected in adipose-derived cancer-associated fibroblasts (AD-CAFs) in vitro were also detectable in mouse pancreatic cancer models: (**a**) Schematic illustration of the KPC xenograft model and treatment protocol. KPC cells were transplanted subcutaneously into C57BL/6 mice, and excised tumors were subjected to metabolomic analysis; (**b**) Representative images of the KPC xenograft tumor. These tumors recapitulate the human pancreatic cancer. Scale bar: 100 μm; (**c**) N8-acetylspermidine was detected in the KPC xenograft tumor; (**d**) Four metabolites detected solely in the AD-CAFs and not in the progenitors were also detected in the KPC xenograft tumor.

Next, we examined whether these AD-CAF-specific metabolites could also be detected in mouse tumor tissues (Figure 7a). Figure 7b displays the tumor tissue section. In this mouse model, mouse cells were spontaneously induced to differentiate into CAFs, as confirmed by the MT staining. This model also contained a population of LIF-positive CAFs. Tumor samples were collected from three independent mice, and metabolomic analyses were performed (Figure 7c,d). N8-acetylspermidine was indeed detected, as in the in vivo analysis, and other metabolites were also detected in living tumor tissues; however, some of these metabolites were close to the detection limit.

## 4. Discussion

### 4.1. Metabolic Transformation in CAFs

In this study, we used an original experimental approach to induce cell differentiation into CAFs in vitro and performed metabolomic analysis to distinguish cancer cells from CAFs. Since the TME contains CAFs in addition with various cell types, such as blood or immune cells, it is difficult to understand the metabolic changes that occur in each cell type. However, in this study, we accurately determined the metabolic transformations that occur in CAFs. Previously, molecular analyses have revealed changes in metabolic genes expressed in CAFs [34,35,36]. These results suggested that CAFs within tumors have an active glycolytic metabolism. In this study, we verified AD-MSCs as the progenitors of CAFs. Furthermore, we compared AD-MSCs and AD-CAFs to precisely elucidate the metabolic changes that occur during differentiation of AD-MSCs into CAFs. It has been shown that CAFs differentiate from MSCs and pancreatic stellate cells (PSCs) by interaction with cancer cells [4,7,8]. Koikawa et al. collected adipose tissue from mice and co-cultured it with mouse pancreatic cancer [37]. Ohlund et al. also co-cultured mouse- or human-derived PSCs with mouse or human pancreatic cancer-derived organoids [7]. On the other hand, we utilized immortalized AD-MSC cell line and pancreatic cancer cell line, and these cell lines could recapitulate CAF heterogeneity and its important functions. Proliferation of cells and tissues derived from living organisms and their properties tend to be unstable; therefore, CAFs derived from such sources are also unstable and challenging to replicate. However, the use of immortalized cells allowed CAFs to maintain their heterogeneity and important functions in the tumor [10,18], making the investigation of CAF- specific metabolites possible in this study. The cancer cell line used in this study was a human pancreatic cancer cell line. However, the robustness and homology of this data needs to be verified in various types of cancer cells, including those derived from various solid tumors, such as gastric cancer and colon cancer. In addition, it is necessary to validate the results of this study using 3D in vitro assays that consider the presence of ECM components that are known to be crucial players in CAF biology.

### 4.2. Polyamine Metabolism in CAFs

Putrescine and N8-acetylspermidine were detected as metabolites specific to CAFs. Putrescine is synthesized from ornithine and is converted to spermidine. In turn, spermidine is converted to N8-acetylspermidine by the histone acetyltransferase P/CAF (KAT2B) in the presence of acetyl CoA. Since the synthesized N8-acetylspermidine is deacetylated by polyamine deacetylase, decreased activity of this enzyme causes accumulation of N8-acetylspermidine in CAFs. However, owing to the high substrate specificity of polyamine deacetylase, it does not deacetylate other acetylspermidines, such as cytoplasmic N1-acetylspermidine or N1-acetylspermine [38]. In addition, selective inhibition of polyamine deacetylase activity in HeLa cells has been demonstrated to increase N8-acetylspermidine levels but not acetylated histone levels. Additionally, polyamine deacetylase has a different function than histone diacetylase (HDAC) [39]. Recently, studies have reported that two Zn^2+^-dependent HDACs, HDAC6, and HDAC10, function as polyamine deacetylases [40,41]. Therefore, we also examined the expression of *HDAC6* and *HDAC10* in AD-MSCs and AD-CAFs but found no changes in their mRNA levels. However, to investigate whether N8-acetylspermidine accumulates in CAFs because of decreased activity of polyamine deacetylase, it is necessary to measure the amount and activity of the enzyme products. Notably, putrescine was upregulated in AD-CAFs, suggesting that N8-acetylspermidine may have been synthesized more by P/CAF via spermidine in the CAFs than progenitors. However, we observed no changes in the mRNA levels of *KAT2B* or *HDAC*. Further studies are needed to determine the pathway of N8-acetylspermidine synthesis and metabolism by performing detailed enzyme activity and metabolite flux analyses.

### 4.3. N8-Acetylspermidine Is a Potential Biomarker of CAF

N8-acetylspermidine was the only AD-CAF-specific metabolite identified in this study. Although the source of N8-acetylspermidine in tumors cannot be determined in vivo by metabolomic analysis, in this study, we speculate N8-acetylspermidine to be CAF-derived. The presence of this metabolite needs to be verified by metabolite analysis of KPC cells. However, since this metabolite was not detected in vitro in human pancreatic cancer-derived cells such as Capan-1 cells, its level may be an indicator of the amount of CAF present in the tumor. Furthermore, we detected this metabolite in a pancreatic cancer mouse model. Since N8-acetylspermidine was not detected in Capan-1 cells, we assumed that N8-acetylspermidine is only biosynthesized in CAFs in vivo. Determination of the source of N8-acetylspermidine in tumors in vivo is challenging for the following reasons. First, there is no antibody available against N8-acetylspermidine. Second, accurate metabolite analysis is difficult in cells in vivo or in direct co-culture because metabolites change with the time required for FACS sorting; identification of CAF subtypes that release N8-acetylspermidine may facilitate identification of the source. Remarkably, N8-acetylspermidine has been detected more frequently in patients with early-stage pancreatic cancer than in that in healthy subjects, and thus is likely to be a promising CAF-specific metabolite [42]. The other metabolites presented in Figure 6 are either biosynthesized in CAFs or taken up by CAFs from Capan-1 cells. We observed macropinocytosis and secretion of extracellular vesicles, such as exosomes, in the TME. Given that putrescine could be synthesized by both Capan-1 and CAF, we presume that putrescine detected in CAFs was either synthesized in CAFs or transported to CAFs from Capan-1 through these intercellular transport mechanisms. At present, while the relationship between micropinocytosis and putrescine is not clear, it would be beneficial to perform block micropinocytosis using pharmacological inhibitors and investigate the impact on putrescine production in the future.

Cancer cells and CAFs generally grow under hypoxia and hypotrophic conditions, while the in vitro experiments were conducted in normoxia. However, the same metabolites were detected as in the in vivo experiments, which is considered to be a hypoxic environment. Therefore, the in vitro experiments may have mimicked some localized hypoxic environments. In the future, it will be necessary to examine the metabolites in more detail by reproducing the hypoxic environment in vitro.

## 5. Conclusions

In this study, we used a simple in vitro experimental system to analyze metabolites with high accuracy and identify several factors that could be potential biomarkers of pancreatic cancer. Although the CAF types produced in this simple experimental system may be limited, the potential biomarkers identified in this study are expected to prove to be important in pancreatic cancer in vivo.

## Data Availability

RNA sequencing data is available at the DDBJ Read Archive under accession number DRR231745–DRR231748. Raw data are available from the corresponding author upon request.

## Supplementary Materials

The following are available online at https://www.mdpi.com/article/10.3390/cancers14061375/s1, Figure S1: Global gene expression pattern showed that AD-MSCs were differentiated into CAF.
