# Supplementary material for: Potential Metabolite Markers for Pancreatic Cancer Identified by Metabolomic Analysis of Induced Cancer-Associated Fibroblasts
Source: Cancers (Basel). 2022 Mar 8;14(6):1375. doi: 10.3390/cancers14061375 (PMC8945883; doi:10.3390/cancers14061375)
Supplement: Supplementary file 1 [file cancers-14-01375-s001.zip › cancers-1582076-supplementary.pdf]

cancers

MDPI

Supplementary material: Potential Metabolite Markers for Pancreatic Cancer Identified by Metabolomic Analysis of Induced Cancer-Associated Fibroblasts

Yoshihiro Miyazaki, Nobuhito Mori, Yuka Akagi, Tatsuya Oda and Yasuyuki S. Kida*

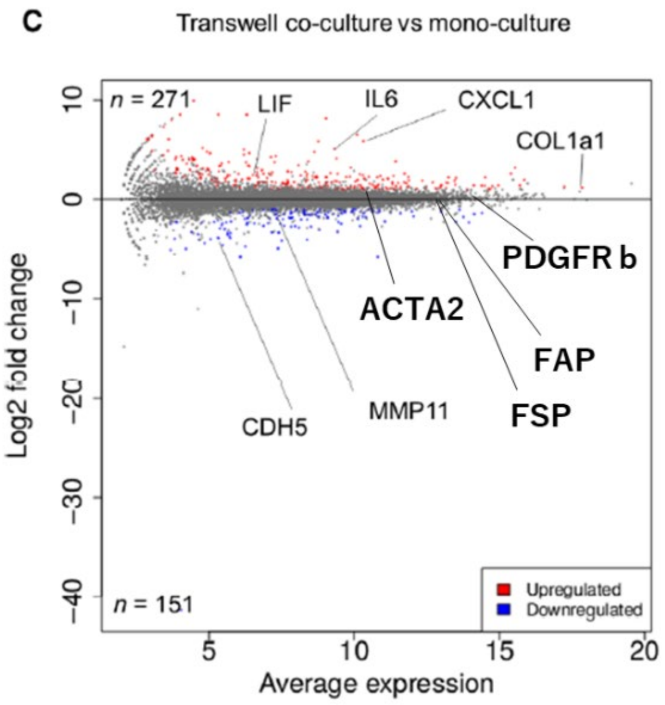

Figure S1: Global gene expression pattern showed that AD-MSCs were differentiated into CAF. MA plot, a scatter plot of log2 fold change versus the average expression, showing differentially expressed genes (adjusted p-value < 0.05 and log2 [fold change] ≥ 1) in transwell co-cultured AD-CAFs compared to those in monocultured AD-MSCs. Upregulated genes are shown in red, and downregulated genes are shown in blue.
